# Enhancing medicine price transparency through price information mechanisms

**DOI:** 10.1186/1744-8603-10-34

**Published:** 2014-05-08

**Authors:** Michael Hinsch, Miloud Kaddar, Sarah Schmitt

**Affiliations:** 1 Expanded Programme on Immunization (EPI), Department of Immunization, Vaccines and Biologicals (IVB), World Health Organization, Avenue Appia 20, Geneva 1211, Switzerland

**Keywords:** Medicine price information mechanisms, Price transparency, Procurement, Access to medicines

## Abstract

**Background:**

Medicine price information mechanisms provide an essential tool to countries that seek a better understanding of product availability, market prices and price compositions of individual medicines. To be effective and contribute to cost savings, these mechanisms need to consider prices in their particular contexts when comparing between countries. This article discusses in what ways medicine price information mechanisms can contribute to increased price transparency and how this may affect access to medicines for developing countries.

**Methods:**

We used data collected during the course of a WHO project focusing on the development of a vaccine price and procurement information mechanism. The project collected information from six medicine price information mechanisms and interviewed data managers and technical experts on key aspects as well as observed market effects of these mechanisms.

The reviewed mechanisms were broken down into categories including objective and target audience, as well as the sources, types and volumes of data included. Information provided by the mechanisms was reviewed according to data available on medicine prices, product characteristics, and procurement modalities.

**Results:**

We found indications of positive effects on access to medicines resulting from the utilization of the reviewed mechanisms. These include the uptake of higher quality medicines, more favorable results from contract negotiations, changes in national pricing policies, and the decrease of prices in certain segments for countries participating in or deriving data from the various mechanisms.

**Conclusion:**

The reviewed mechanisms avoid the methodological challenges observed for medicine price comparisons that only use national price databases. They work with high quality data and display prices in the appropriate context of procurement modalities as well as the peculiarities of purchasing countries. Medicine price information mechanisms respond to the need for increased medicine price transparency and have the potential to contribute to improved access to medicines in developing countries.

Additional research is required to explore more specific aspects. These include the market effects of dedicated donor funds for certain medicines to explain the driving force of user demands, and the effects of increased price transparency on different groups of medicines in context of the maturity of their markets.

## Background

Opinions diverge about the effects of improved price transparency in medicine markets. There is some debate as to whether increased price transparency would have more positive or negative effects on the availability and affordability of medicines [1].

Medicine price information mechanisms are significant tools to achieve increased price transparency by providing information on product prices as well as the components that influence these prices, such as volume and product quality. Information provided by such mechanisms allows developing countries^a^ to make more informed decisions about the most efficient and effective ways to procure medicines. In addition to providing comparative price information between market participants, which would be challenging to access in similar quality for individual countries without such tools, medicine price information mechanisms can help identify and improve inefficiencies in existing procurement systems when providing contextual information about the reported prices.

Cost reductions through improved efficiencies have the potential to create more effective public health systems in developing countries, where the pricing of medicines is a critical factor in accessing treatment. This article takes a closer look at the pros and cons of increased medicine price transparency with regards to access to medicines in developing countries and assesses the role of medicine price information mechanisms in this context.

It is important to note that any comparison of medicine prices will only produce significant results when taking into account the quality aspect and comparing products of same and similar effect. Unregulated, mislabeled or deliberately counterfeited pharmaceutical products pose a significant problem not only to procurement agencies but also to the safety of patients. Therefore it is important to limit the comparison of medicine prices to generic products that are considered identical ‘to a brand name drug in dosage form, safety, strength, route of administration, quality, performance characteristics and intended use [2]’.

### Medicine prices and access to health care in developing countries

There are various factors influencing the access to medicines for the public sector, including the availability of sustainable financing, reliable health systems, rational selection and use of medicines, as well as affordable pricing [3]. Medicine prices form one important aspect determining access to affordable health care in developing countries, where systems are often not able to provide sufficient appropriate treatment and care. Patients frequently need to pay for medication as part of public sector treatment or are required to purchase medicines in the private sector due to low availability in the public health system [4]. These payments can be significant. WHO statistics reveal that in 32 percent of the Low and Lower middle income countries^b^ private expenses constitute more than half of the total health spending [5]. Furthermore, statistics show that ‘in countries where patients pay for medicines in the public sector, average prices of generic medicines range from 1.9 to 3.5 times international reference prices’ between different WHO regions [6].

Price differences between regions and countries depend on a variety of factors and need to be understood in their respective context. WHO identifies some of the contributing factors as shortcomings in public procurement systems, insufficient financing of public health care systems and other policy related issues [6]. Lower medicine prices can be achieved by addressing some of the inefficiencies through strategies such as bulk purchasing, increased competition, skillful negotiation and thorough supply management [7].

Increased transparency of medicine prices can have a positive impact on the application of such strategies for countries that have not reached their full potential in achieving appropriate prices and fully efficient procurement systems.

### Medicine price components

The meaningful comparison of international medicine prices requires an understanding of the various components constituting a price. In addition to varying market conditions for individual medicines, such as ease of market access and level of competition, specific procurement conditions can have additional effects on prices. Among other factors, these can include volumes purchased, the terms of delivery (Incoterms) applying to a contracted price and the currency of the purchase agreement. Contracts may also include specific conditions such as bundling or special discounts [4].

While some purchase agreements are made directly with medicine suppliers and manufacturers, others are made through procurement agents, local import agents, wholesalers or distributors whose mark-ups need to be taken into account as well. Manufacturer prices are often kept confidential [4], therefore conducting reliable and useful medicine price comparisons can be challenging for countries without appropriate skills and access to additional information. Morgan et al. [8] note several disadvantages of confidential rebate agreements between countries and pharmaceutical companies, such as increased administrative costs and a loss of transparency which makes it more difficult to obtain comparative price information. They advise policy makers to achieve more transparent prices and contract structures in order to address disproportionate price differences within and between countries.

### Call for increased price transparency

In 2001, the World Health Assembly passed Resolution WHA 54.11, outlining the WHO medicines strategy [9]. The resolution focused on the expansion of access to essential drugs and the promotion of equitable access to medicines. In addition to other aspects, the resolution called for action to increase medicine price transparency by exploring ‘the feasibility and effectiveness of implementing, in collaboration with nongovernmental organizations and other concerned partners, systems for voluntary monitoring drug prices and reporting global drug prices with a view to improving equity in access to essential drugs in health systems, and to provide support to Member States in that regard’ [9].

In the years following the resolution, various medicine price information mechanisms were instituted, including five of those described in this paper, targeted primarily at developing countries and with the aim of increasing price transparency.

### Effects of increased price transparency

Increased price transparency can have various effects on the medicine market. In competitive markets, economic theory predicts a decrease and convergence of prices as a consequence of improved transparency. Measures undertaken in high income countries to increase price transparency in private medicine markets have produced this effect [10].

There is only a small amount of evidence of the net effects of price transparency in developing country markets [1]. However, advocates of increased price transparency highlight potential cost savings through better informed procurement agents and efficiency improvements for established procurement systems [4]. The availability of comparative pricing information from other buyers allows purchasing countries to leverage their negotiation position and demand more favorable prices for their purchases [1]. Improvements in efficiency can result from reduced search costs [11] and from benchmarking national medicine prices against the results achieved by other countries. Exploring the effects of increased price transparency of medical devices, Pauly and Burns [12] note how comparing the procurement results of different hospitals can help improve the performance of staff. Similar effects can be expected for the performance of national procurement agencies.

However, these effects do not necessarily translate into cost savings or other effects that may result in increased access to medicines for developing countries. As Hviid and Møllgaard [13] show, medicine suppliers may adapt their price strategies to the improved knowledge of buyers and refrain from offering lower prices or individual discounts to countries. Critics point out that this can lead to a convergence of prices to a more uniform level. In equitable pricing scenarios, this could jeopardize the benefits the poorest countries might derive from price discrimination as they would eventually need to pay more for their medicines if prices became more uniform [14].

Other potential negative effects of price transparency include possible harmful consequences for medicine manufacturers that could also affect the availability of medicines in developing countries. As Outterson [15] notes, critics maintain that reduced medicine prices due to increased price transparency could harm profits to an extent that companies may consider withdrawing their product from the market or reducing their investment in the development of new drugs specific to the needs of developing countries. Pauly [16] indicates that profit maximizing companies invest in research and development based on the expected demand and value of a product that makes it reasonable to incur such a financial risk. However, should profits fall below a certain margin, such investments may indeed be cancelled. There may also be the risk of market exit of companies whose cost structure cannot compete with more efficient competitors, which in turn could negatively affect developing countries’ access to medicines.

Different effects of increased transparency can be expected in markets with different stages of maturity. For example, in less mature oligopolistic markets, which would be a characteristic of innovative medicines, given demand for a product in the absence of an abundance of suppliers will result in high prices. However, as Møllgaard and Overgaard [17] note, varying market behavior of suppliers and buyers can lead to different outcomes. While increased price transparency could provide incentives for collusion between suppliers and therefore drive prices up, transparency can also lead to increased competition, provided that market behavior of buyers has previously been constricted due to limited information.

It could therefore be acknowledged that increased price transparency can trigger both positive and negative consequences for a developing country’s access to medicines. As noted by Møllgaard and Overgaard [17] regarding market effects of increased price transparency in oligopolies, ‘the effect of information or transparency needs to be assessed on a case-by-case basis’. Additional research and a more detailed analysis on the net costs or benefits of increased medicine price transparency will be necessary to identify the specific effects in various market scenarios with different levels of maturity.

### Medicine price information mechanisms

Van Dongen [18] conducted a review of 50 national and 21 international price sources, and provided an overview of the availability of medicine price information as well as an evaluation of the reported information. Her analysis came to the conclusion that despite the wide availability of medicine price information websites large variations between medicine prices of different countries still exist for identical products^c^. Prior to the establishment of most of the mechanisms reviewed, Danzon and Chao [19] showed that earlier comparisons of medicine prices between countries were not methodologically sound. Unrepresentative sampling and different approaches in the collection of national drug prices resulted in limited comparability between different data sets. The misinterpretation of data could therefore have led to wrong conclusions that did not allow for improvements of existing national procurement strategies.

Comparability of prices between countries is often limited due to differences in procurement modalities and supply chain systems. Additional factors may be in place that make a market particularly attractive for medicine suppliers (e.g. market size and disease burden) or allow a country access to prices attributable to donor support (e.g. per capita income). Other barriers affecting the correct interpretation of data exist, such as differences in reporting languages. However, van Dongen [18] also identified significant price discrepancies between rather similar countries, indicating considerable opportunities for further improvements.

From a methodological point of view, van Dongen’s study [18] concluded that international price comparisons are best conducted utilizing mechanisms that contain price information from multiple countries. This is considered to be less time consuming and error-prone than retrieving data from multiple national websites which may apply different methodologies for data collection [18]. Information provided about the procurement systems and procedures used to achieve individual prices further contributes to meaningful results of price comparisons.

## Methods

Original research was conducted for the World Health Organization’s (WHO) Vaccine Product, Price and Procurement (V3P) project [20]. The V3P project intends to address the need of many Middle income countries that are not benefitting from GAVI support and are facing challenges in the sustainable introduction of new and essential vaccines. The project focuses on the provision of accurate, reliable and neutral vaccine product, price and procurement data, which are critical for the forecasting, budgeting and sustainable financing of vaccines, yet currently not available for Middle income countries. The intended output of the V3P project is an information mechanism that allows for increased price transparency and more informed decision making in the vaccine implementation and procurement processes of Middle income countries. One of the work streams of the project included the review of six medicine price information mechanisms to explore existing tools and lessons learned through the operation of price information mechanisms: WHO’s Global Price Reporting Mechanism (GPRM) [21], WHO/Health Action International’s (HAI) medicine price database [22], Management Sciences for Health’s (MSH) International Drug Price Indicator Guide [23], WHO/Western Pacific Regional Office’s (WPRO) Price Information Exchange (PIE) [24], the Global Fund’s Price and Quality Reporting (PQR) tool [25], as well as Médicins Sans Frontières’ (MSF) Untangling The Web (UTW) report [26]. These mechanisms were selected to provide information on best practices and lessons learned during the set-up and operation that should inform a potential vaccine price information mechanism envisioned by the V3P project.

The review was conducted through direct test utilization of the mechanisms and the examination of supporting documents to learn about the mechanisms’ functionality and their utility as data sources. Furthermore, 14 guided semi-structured interviews were conducted with data managers and other representatives of organizations hosting these mechanisms in course of the V3P project in 2011 and 2012 in order to learn about the evolution of the mechanisms, challenges encountered, identified best practices and user feedback. Certain findings of this review are relevant for the debate on price transparency and are presented in this article.

## Results

### Setup and functionality of the reviewed mechanisms

The six medicine price information mechanisms reviewed for the V3P project include international price data with five of the mechanisms also providing information on the procurement modalities that resulted in the reported prices. Table 1 provides an overview of the objective, target audience and information provided by GPRM, MSH’s Guide, PIE, PQR, UTW and WHO/HAI’s price database.

**Table 1:** An overview of selected medicine price information mechanisms

**Mechanism (host, year of inception)**	**Scope and focus**	**Information provided**			**Objective**	**Target audience**
		**Product**	**Pricing**	**Procurement**		
GPRM* (WHO, 2005)	38 items of HIV/AIDS products with different strengths (data on malaria and tuberculosis medicines will be reintroduced after completion of quality control). About 70,000 entries of transactional data.	Product group, strength, dosage form, manufacturer, country of manufacture, generic name, brand name, type of package, base unit of measure, quantity per package, number of packages, total number of smallest unit.	Unit price per smallest unit, total price paid.	Procuring country, date the order was placed, delivery date, customer name, Incoterms, shipment method, payment terms, consignee name.	To promote medicine price transparency by collecting, consolidating, analyzing and disseminating strategic information regarding affordability, accessibility, and availability of HIV, tuberculosis and malaria medicines and diagnostics, and to inform the decision making process of procurement agencies working at national and global levels.	National and global procurement agencies, donor agencies and NGOs.
PQR* (The Global Fund, 2004)	Circa 115 items of HIV/AIDS, malaria and tuberculosis medicines and other health products (about 35,000 entries of transactional data).	Product, strength, dosage form, manufacturer, supplier, generic name, type of package, base unit of measure, quantity per package, number of packages.	Pack price, unit price, total price paid; limited information about mark-ups.	Procurement agents, procurement date, scheduled and actual delivery date, Incoterms; information about freight and insurance is provided as an estimate.	PQR is a grant monitoring tool for the Global Fund and its donors. It provides information on the efficiency of grant utilization, monitors consistency of costs with international reference ranges, and follows up on compliance with quality assurance policies.	Principal Recipients of Global Fund medicine support as well as the Global Fund secretariat and partners.
**International Drug Price Indicator Guide (MSH, 1986)**	About 1,100 medicines and other health products, focusing on products included in WHO’s Essential Medicines List (EML).	Generic name, strength, dosage form, route of administration, WHO status, defined daily dose, Anatomic Therapeutic Chemical (ATC) classification.	Buyer and supplier prices: pack price and unit price, with indication of highest, lowest and median listed price; approximated shipping costs for suppliers’ listed prices.	Incoterms.	To make price information more widely available in order to assist in planning budgets and help facilitate the procurement of pharmaceuticals and other health technologies of assured quality (as declared by data providers without constituting pre-qualification or quality approval from MSH or WHO) for developing countries for the lowest price possible, thereby contributing to the equitable access to health.	Ministries of Health, national medical procurement agencies and other institutions responsible for medicine procurement.
**PIE (WHO/WPRO, 2009)**	40 medicines (only 2009–2010 data available).	Generic name, brand name, dosage form and strength, package size, volume procured, manufacturer and supplier.	Pack price, unit price, indication about insurance and freight costs.	Procurement method and date; description of countries’ public medicines procurement system; regulations and quality assurance; taxes on medicines; medicines financing/expenditure and pricing policies.	To provide WPRO member states with a tool to help them assess their procurement efficiency over time and leverage their negotiation power with medicine suppliers, in order to obtain the best possible prices.	Ministries of Health, national and national medical procurement agencies.
**UTW (MSF, 2001)**	33 ARV medicines.	Therapeutic class, manufacturer, generic name, brand name, strength, dosage form, quality assurance (WHO prequalification and US FDA tentative approval status), WHO guidelines, updates on ARV developments, patent status of registered products.	Price information is provided by product formulation and daily dosage. Indication of market trends (i.e. evolution of lowest generic price and innovator price for middle and low income countries). Access eligibility criteria to certain prices for specific countries and organizations.	Indications of Incoterms provided by ARV manufacturers; world sales of originator product.	To provide a transparent platform of price information made available by medicine suppliers on the prices they offer to different categories of countries and organizations for the benefit of countries purchasing ARV medicines and receiving support from organizations involved in the provision of ARV drugs to beneficiaries in need.	Purchasers of ARV drugs who buy the products for use in developing countries, implementers of HIV programs, civil society and policy makers in public health and intellectual property.
**WHO/HAI Database (HAI, 2003)**	Information derived from about 100 surveys providing price data on a selection of 50 medicines (including 14 pre-selected global products, 16 pre-selected regional products, and 20 products selected by the individual research groups).	Manufacturer, generic name, brand name, strength, dosage form, unit of measure, national EML status.	Government procurement price and final patient price for the public, private, and other sectors in USD, local currency and as a ratio to an international reference price. Affordability of standard treatments. Medicine price components in the supply chain (manufacturer’s selling price, insurance and freight; landed price; wholesale selling price for the private sector, or the price indicated by central medical stores for the public sector; retail price for the private sector, or the dispensary price for the public sector; dispensed price).	N.a.	To improve the availability and affordability of essential medicines through the collection, analysis and publication of medicine price, availability, affordability and medicine price component data across health-care sectors and regions in a country. To improve price transparency. To provide information on pricing policy options and monitoring the impact of such policies.	For survey methodology and results: ministries of health, civil society, academia and others. For policy advice: national decision makers in health policy.

*GPRM and PQR are listed in sequence due to the similarity of the two information mechanisms. Otherwise the mechanisms included in this table are listed in alphabetical order.

All six mechanisms include the goal of increased price transparency for their various target audiences in the fields of policy making, public health budgeting or public medicine procurement. With GPRM, the WHO/HAI database and PIE, WHO has been directly involved in the development and operation of three of these mechanisms. Since 2000, WHO also collaborates with MSH on the International Drug Price Indicator Guide.

### Information provided

All mechanisms are publicly accessible and provide information on product characteristics and pricing. With the exception of WHO/HAI, the mechanisms also include information about procurement modalities realized by national procurement agencies.

GPRM and PQR both contain transactional data of medicines and other health products focusing on HIV/AIDS, malaria and tuberculosis diagnosis and treatment. GPRM was developed from an earlier version of PQR and still shares similarities, although both mechanisms have evolved differently. UTW, which is part of MSF’s Campaign for Access to Essential Medicines, focuses on Anti-Retroviral (ARV) medicines only. MSF considers UTW a provider of complementary data to information provided by GPRM and PQR.

### Information sources

GPRM is built on a broad collaboration of several partners and donors and is hosted at WHO. The mechanism encourages competition and accountability of suppliers and promotes benchmarking of supplier prices. Established relationships with specific procurement agencies as regular data providers allow GPRM to receive a constant flow of data. One of GPRM’s data providers is the Global Fund through its PQR mechanism. Data included in the PQR is automatically fed into GPRM^d^. PQR receives its information from Principal Recipients of Global Fund support who are required to provide price information following the receipt of goods purchased with Global Fund support. These prices are used to monitor the Principal Recipients and ensure that their procurement concurs with Global Fund standards and is within the international average price ranges.

UTW sources its information from ARV manufacturers who voluntarily provide price and procurement information on an annual basis. This makes UTW the only source of medicine price information reviewed that includes information provided by pharmaceutical suppliers to identify criteria for price differentiation between buyers.

PIE, MSH’s Guide and WHO/HAI’s price database contain a broader range of medicines. PIE provides comparative information on the procurement prices of selected medicines that focus on common conditions and are widely used by countries participating in the mechanism. The mechanism receives its data from member states who voluntarily provide their price and procurement information on an annual basis. MSH’s International Drug Price Indicator database provides product, price and procurement information on various health items, including medicines that are registered in WHO’s Essential Medicines List (EML). Data providers include NGOs, international donor agencies, UN agencies and purchasing countries.

Unlike the other five mechanisms reviewed, WHO/HAI collects its data from independent researchers who conduct medicine price surveys according to the instructions outlined in WHO/HAI’s survey manual^e^. A typical survey collects data on 50 medicines in six geographic or administrative areas of a country and samples public health facilities, registered private retail medicine outlets and up to two other sectors in which medicines are commonly assessed (such as the mission sector). In addition to providing access to survey data, WHO/HAI provides advice on national pricing policy options and gives instructions on the establishment of monitoring systems through the WHO/HAI manual.

Depending on the type of data source, the reliability of data comparison between countries may vary. Data collected by mechanisms that source their information from countries does not reveal the number of middlemen and the amount of mark-ups included in the reported price. More reliable results from medicine price data comparisons between countries can be expected from mechanisms that source their data directly from the manufacturer (UTW), from international procurement agents (GPRM), or from data sources with a strong incentive to cut avoidable mark-ups (PQR and the Principal Recipients of Global Fund support).

### Data volume

GPRM and PQR handle large amounts of data every year. Since their inception, GPRM and PQR have collected around 70,000 and 35,000^f^ entries of transaction data, respectively. In comparison, MSH’s database provides product, price and procurement information on more than 1,100 items, including health products other than medicines. PIE provides information about 40 medicines purchased by 23 member states within WHO’s Western Pacific Region; however, only for the years 2009 and 2010. The latest UTW reported price information on 33 ARV medicines [27]. WHO/HAI depends on the completion of individual country surveys to build its database. To date, the WHO/HAI database provides information on 400 entries of various presentations of medicines from surveys conducted in 54 countries, with individual countries being the focus of one or more of these surveys.

### Data collation

All mechanisms except UTW and WHO/HAI allow for the automatic collation of medicine price data between different countries and years. GPRM, PQR and PIE break down price information into more detail and thereby enable users to conduct additional price analyses. UTW is available in report format only and while price developments of individual medicines over time are depicted in charts, comparisons between years need to be conducted manually between different reports. International price comparisons conducted with WHO/HAI data are limited to the availability of survey data for specific countries and years.

The comparability of international medicine price data requires certain modifications to account for differences in purchasing power parity or inflation when comparing across countries or years. The WHO/HAI manual [4] provides detailed instructions for the users of its database to make such modifications and allow for the compilation of meaningful data. MSH’s Guide indicates exchange rates used for individual years and instructs its users to take into account additional factors such as financing, procurement modalities and supply chain peculiarities that may influence price calculations. Data included in the GPRM, PQR, PIE and UTW mechanisms are presented in USD as indicated by the individual data providers which simplifies collation but also inhibits the risk of potential calculation errors made before the data reaches the database of the information mechanisms.

### Observed effects of medicine price information mechanisms

Interviews conducted with data managers of the reviewed price information mechanisms indicated certain market effects that may be connected to the operation of some of the mechanisms.

PIE learned from some of the countries participating in the mechanism that they were able to negotiate more favorable terms for their procurement contracts after having had access to price information from other countries in Pacific region which paid significantly less for their medicines. Similarly, a 2004 user survey conducted by MSH indicated that almost half of the price comparisons conducted with information from the International Drug Price Indicator Guide contributed to some form of price savings. WHO/HAI observed changes in national pricing policies that have resulted in lower prices in Indonesia, Lebanon and elsewhere [4].

Mandatory reporting requirements applied to Principal Recipients of the Global Fund most likely contributed to an increased demand for high quality products. This effect was observed by PQR data managers following the introduction of compulsory reporting of price and procurement details for all goods purchased with Global Fund support to PQR. Although the Global Fund always required the purchase of high quality products, the constant and immediate monitoring of purchases through the PQR triggered a significant increase in adherence to procurement and supply management requirements and an increase in efficiency.

Since its launch, UTW has achieved a lot of recognition on the ARV market, providing an incentive to manufacturers of ARV drugs to voluntarily and regularly provide price information to this annual price report. The report now includes information from all major ARV manufacturers that provide originator brands of products recommended by WHO treatment guidelines. The team working on MSF’s UTW mechanism observed that prices for some of the generic ARV drugs included in their report have decreased and converged over time. This effect was observed particularly for medicines produced by more than three generic companies, which includes increased competition in addition to improved price transparency as a potential cause of price decreases. Based on the experience with UTW, MSF notes a positive effect of price transparency on increased access to medicines.

## Discussion

Established for varying target audiences, the reviewed mechanisms respond to the need for more transparent price information expressed by countries, donors and various other entities. There is little data available on how many countries are utilizing the reviewed mechanisms. Still, there is continuing demand for medicine price information. While not all of the reviewed information mechanisms conduct systematic user evaluations, MSH’s Guide, PIE and UTW receive positive feedback from the users of their data. GPRM and PQR regularly respond to user requests regarding their data and mechanism. At the same time WHO/HAI continues to provide technical support to researchers using WHO/HAI methodology and receives requests to use the data in their database. Following the launch of the manual, WHO/HAI observed an unexpectedly high demand for training workshops on the methodology. To date, about 100 surveys using WHO/HAI’s methodology have been completed or are nearing completion.

The reviewed mechanisms allow for the comparison of international medicine prices while avoiding methodological challenges observed for comparisons using only national price databases as information sources. GPRM, MSH’s Guide, PIE, PQR and UTW allow users to access information on international medicine prices on a single platform. Data is coherently collected with methodologies and procedures established by the individual mechanisms. WHO/HAI enables price comparisons through use of a standardized methodology and adjustment procedures that allow for the comparison of medicine prices across countries and years. However, one needs to recognize the caveat that all reviewed mechanisms still need to rely on the accuracy of data received from their various sources, over which they have little to no influence.

The majority of the reviewed mechanisms provide some procurement information in addition to pricing data, although to different extents. Such additional information is important when conducting international price comparisons, in order to understand prices in their individual contexts and to determine the types and levels of mark-ups included at different stages of the supply chain. Compared to the other reviewed mechanisms, GPRM and PQR offer the most detailed information on procurement modalities accompanying the reported prices. PIE provides detailed country profiles that present background information on regulations and procedures in place, which enables a better interpretation of price information. MSH’s Guide urges their users to interpret prices in their individual contexts and UTW provides terms of access to certain prices for specific purchasers. WHO/HAI does not provide information on procurement modalities.

### Focus on ARV prices

GPRM, PQR and UTW focus on HIV/AIDS related medicines (with GPRM and PQR also including information on malaria and tuberculosis medicines and related products). The amount of price information available for ARV medicines and other health products focusing on HIV/AIDS is remarkable and may reflect the prominence of HIV/AIDS on the international health agenda over the past decade.

Other increasingly important pharmaceutical segments have not seen the establishment of similar mechanisms with a specific focus. For example, in some Low income and Middle income countries the cost of drugs treating chronic diseases can represent a significant share of public and private medical expenditures [28]. The successful operation of price information mechanisms focusing on HIV/AIDS medicines may provide an incentive for similar tools to be implemented, that focus on other important diseases and public health cost drivers.

The focus on ARV prices of some of the observed mechanisms may also have contributed to the decline of prices of certain medicines reported by data managers. In addition to UTW, the data managers of GPRM and PQR observed similar developments occurring during the operation of price information mechanisms focusing on ARV prices and other products. Nevertheless, these price declines may be due to a variety of causes, potentially including increased price transparency. For example, a strong donor focus and the availability of donor funds [29] created a considerably large and risk-free environment that sets aside ARV from other medicine markets. Additional research would be necessary before drawing any definite conclusions on the causes of the price decreases observed.

### Potential benefits of medicine price information mechanisms

Medicine price information mechanisms contribute to increased price transparency by allowing users to compare prices, understand the composition of these prices and provide better insights into existing procurement systems. Access to increased information can contribute to cost savings and potentially increase the access to medicines for developing countries. In addition to the extraction of price data, the utilization of medicine price information mechanisms may also help countries gain a better understanding of the different components involved in medicine procurement. One important lesson learned from PIE’s experience is that participating countries voluntarily shared their price and procurement information with other countries providing information to the mechanism. This allowed them to evaluate their procurement procedures and compare them to the systems used by other countries.

In order to derive the highest possible benefits from improved price transparency, countries first need to be aware of the existence of tools providing detailed price information. Raised awareness could help increase the utilization of price information mechanisms. Furthermore, clear instructions and user support regarding the most effective utilization of the mechanisms and the correct interpretation of data are necessary to achieve meaningful results. Finally, depending on the need of individual countries, targeted capacity building can help maximize the effects derived from increased information and help improve the access to medicines for developing countries.

## Conclusion

Working with accurate and contextually rich data, medicine price information mechanisms have the potential to play an important role in making medicines prices more transparent. Positive user feedback and ongoing data requests received by the reviewed mechanisms indicate a high demand for increased medicine price transparency. Taking into account the amount of information gathered and accessed by mechanisms that focus on medicines and health products related to HIV/AIDS, malaria and tuberculosis, it would be interesting to see whether and to what extent mechanisms focusing on different disease segments could encounter a similar demand. Market impact assessments exploring the effects of dedicated donor funds may help explain the driving force of user demands.

The divergent views on whether increased medicine price transparency generates more value or harm to the availability and affordability of medicines in developing countries need to be considered within the context of individual medicines. From a theoretical point of view, the effects of increased price transparency are highly dependent on the degree of competition prevalent in a market. Practically speaking, this requires more detailed analyses of different groups of medicines, depending on the maturity of their market.

Additional research would also be necessary to provide more clarity on the extent of which the information and data provided by the reviewed price information mechanisms contributes to price decreases and other market effects. Information gathered during the review of the six medicine price information mechanisms derives from user statements and observations made by data managers on price developments over time. Longitudinal studies building on market analyses and targeted user evaluations may help produce more significant data that may allow conclusions on the impact of information mechanisms on medicine suppliers and purchasing countries.

Nevertheless, as demonstrated through the information provided by the interviewees, the review of the mechanisms did reveal positive effects on the behavior of their users. While the cause(s) of the observed reduced procurement costs for ARV and other medicines still need to be fully determined, countries using the PIE mechanism were able to negotiate more favorable prices for their medicines. Also, the utilization of the PQR mechanism led to the procurement of better quality medicines.

Taking into account these positive effects for developing countries and their health systems, the operation and utilization of medicine price information mechanisms should be encouraged and supported where needed.

### Endnotes

^a^Following the target audience of the medicine price information mechanisms discussed in this article, the authors chose the term “developing countries” to include all countries not defined as High income countries following the World Bank Atlas method (GNI < USD 12,615 per year).

^b^2 out of 36 Low income countries and 17 out of 54 Lower middle income countries (using the World Bank Atlas method).

^c^Van Dongen’s analysis focused on the medicine prices of seven of the 14 medicines collected globally in WHO/HAI surveys.

^d^When this article was written, GPRM only contained data about HIV/AIDS related products, with information about malaria and tuberculosis related medicines and health products undergoing quality control.

^e^The WHO/HAI survey methodology has been published as a manual with an accompanying automated Excel workbook for data entry and analysis. The methodology’s second edition was published in 2008.

^f^The currently available upgraded version of PQR contains about 20,000 entries.

## Competing interests

The authors declare that they have no competing interests.

## Authors’ contributions

MH, MK and SS conceptualized the research. MH and SS reviewed the information mechanisms and conducted interviews. MH prepared the first draft of the manuscript. MK and SS critiqued the draft and added text. Subsequent revisions were made by all authors. All authors reviewed the final draft and approved it for submission.

## References

[B1] Kyle MK Ridley DB Would greater transparency and uniformity of health care prices benefit poor patients? Health Aff 2007 26 5 1384 1391 10.1377/hlthaff.26.5.1384 17848449

[B2] U.S. Food and Drug Administration Generic drugs: questions and answers http://www.fda.gov/drugs/resourcesforyou/consumers/questionsanswers/ucm100100.htm. (Accessed 28 June, 2013)

[B3] World Health Organization More Equitable Pricing for Essential Drugs: What do we Mean and What are the Issues? Background Paper for the WHO-WTO Secretariat Workshop on Differential Pricing and Financing of Essential Drugs: 8–11 April 2001 2001 Geneva, Switzerland: Høsbjør

[B4] World Health Organization, Health Action International Measuring Medicine Prices, Availability and Price Components 2008 2 Geneva, Switzerland: World Health Organization

[B5] World Health Organization World Health Statistics 2012 2012 Geneva, Switzerland: World Health Organization

[B6] World Health Organization The World Medicines Situation 2011. Medicines Prices, Availability and Affordability 2011 Geneva, Switzerland: World Health Organization

[B7] Management Sciences for Health, World Health Organization International Drug Price Indicator Guide 2010 2010 Cambridge MA, USA: Management Sciences for Health

[B8] Morgan S Daw J Thomson P International best practices for negotiating ‘reimbursement contracts’ with price rebates from pharmaceutical companies Health Affairs 2013 32 4 771 777 10.1377/hlthaff.2012.1268 23569058

[B9] World Health Assembly54th World Health Assembly: WHO medicines strategy: 21 May 2001 2001 Geneva; Switzerland: World Health Organization

[B10] Austin AD Gravelle J Does Price Transparency Improve Market Efficiency? Implications of Empirical Evidence in Other Markets for the Health Sector CRS Report for Congress, RL34101, 24 July 2007 2007 Washington, D.C.: U.S: Congressional Research Service

[B11] Stigler GJ The economics of information J Pol Econ 1961 69 3 213 225 10.1086/258464

[B12] Pauly MV Burns LR Price transparency for medical devices Health Affairs 2008 27 6 1544 1553 10.1377/hlthaff.27.6.1544 18997210

[B13] Hviid M Møllgaard HP Countervailing power and price transparency Scand J Econ 2006 108 3 499 512 10.1111/j.1467-9442.2006.00468.x

[B14] Ridley DB Price differentiation and transparency in the global pharmaceutical marketplace Pharmacoeconomics 2005 23 7 651 658 10.2165/00019053-200523070-00002 15987224

[B15] Outterson K Pharmaceutical arbitrage: balancing access and innovation in international prescription drug markets Yale J Health Policy Law Ethics 2005 5 1 193 292 15742578

[B16] Pauly MV Commentary. Drug and vaccine pricing and innovation: what is the story? Managerial Decis Econ 2007 28 407 413 10.1002/mde.1351

[B17] Møllgaard P Overgaard PB Transparency and Competition Policy The Pros and Cons of Information Sharing 2006 Stockholm, Sweden: Swedish Competition Authority 101 129

[B18] van Dongen S Websites Reporting Medicine Prices: A Comparative Analysis 2010 Geneva, Switzerland: World Health Organization in assoc. with Department of Pharmaceutical Sciences, Utrecht University, the Netherlands

[B19] Danzon PM Chao L Cross-national price differences for pharmaceuticals: how large, and why? J Health Econ 2000 19 159 195 10.1016/S0167-6296(99)00039-9 10947575

[B20] Kaddar M New project to provide lower-middle and middle-income countries with up-to-date product, price and procurement information Global Immunization News 2011 http://www.who.int/immunization/GIN_August_2011.pdf (Accessed 17 April 2013)

[B21] World Health Organization Global price reporting mechanism http://apps.who.int/hiv/amds/price/hdd. (Accessed March 15, 2013)

[B22] Health Action International Medicine prices, availability, affordability & price components http://www.haiweb.org/medicineprices. (Accessed March 15, 2013)

[B23] Management Sciences for Health International drug price indicator guide http://erc.msh.org/priceguide (Accessed March 15, 2013)

[B24] World Health Organization, Western Pacific Region Price information exchange for selected medicines in the western pacific region http://www.piemeds.com (Accessed March 15, 2013)

[B25] The Global Fund Price and quality reporting http://www.theglobalfund.org/en/procurement/pqr (Accessed March 15, 2013)

[B26] MSF Access Campaign Untangling the web of antiretroviral price reductions http://utw.msfaccess.org. (Accessed March 15, 2013)

[B27] Médecins Sans Frontières Untangling the Web of antiretroviral price reductions http://d2pd3b5abq75bb.cloudfront.net/2012/11/27/10/34/06/884/MSF_Access_UTW_15th_Edition_2012_updatedOct2012.pdf (Accessed April 17, 2013)

[B28] World Health Organization Global Status Report on Noncommunicable Diseases 2010 Geneva, Switzerland

[B29] Waning B Kaplan W King AC Lawrence DA Leufkens HG Fox MP Global strategies to reduce the price of antiretroviral medicines: evidence from transactional databases Bull World Health Org 2009 87 520 528 10.2471/BLT.08.058925 19649366 PMC2704041

